# Bone mineralization and the effects of elevated osteopontin: from symmetry-breaking foci to 3D space-filling tessellation†

**DOI:** 10.1039/d5fd00013k

**Published:** 2025-03-12

**Authors:** Joseph Deering, Daniel J. Buss, Roland Kröger, Hojatollah Vali, Maureen J. Lagos, Natalie Reznikov, Marc D. McKee

**Affiliations:** a Faculty of Dental Medicine and Oral Health Sciences, McGill University Strathcona Anatomy and Dentistry Bldg, Rm M73 3640 University Street Montreal QC H3A 0C7 Canada marc.mckee@mcgill.ca; b Department of Anatomy and Cell Biology, School of Biomedical Sciences, Faculty of Medicine and Health Sciences, McGill University Montreal QC H3A 0C7 Canada; c Department of Physics, University of York York YO10 5DD UK; d Department of Materials Science and Engineering, McMaster University Hamilton ON L8S 4L8 Canada; e Department of Bioengineering, Faculty of Engineering, McGill University Montreal QC H3A 0E9 Canada

## Abstract

At the nanoscale, lamellar bone tissue mineralization ensues *via* heteronucleation of small mineral foci within the osteoid. The foci grow to produce a mature, volume-filling tessellation pattern at the micrometer-scale. Mineralization-inhibiting osteopontin (OPN) mediates this bone mineralization pathway and, eventually, the microscale properties of bone tissue. Using 2D and 3D electron microscopy, here we have assessed how the abundance of OPN can affect nanoscale mineralization, mineral ripening, and microscale patterning of mineral in normal wild-type mouse bone, and we compare that to mutant mouse models having elevated OPN (*Fgf23*^−/−^ and *Hyp* mice). When OPN is elevated, volume-filling mineral tessellation was incomplete (showing a four-fold increase in mineral surface area in the vicinity of the mineralization front in *Hyp* bone). Immunogold labeling showed excessive OPN in the foci, suggesting an arrest of their growth and an interruption of the pathway towards microscale tessellation. In *Fgf23*^−/−^ mice, electron tomography and 3D focused ion beam–scanning electron microscopy (FIB-SEM) imaging of mineral foci show instances of core–shell morphology with crystalline mineral confined to the focus interior, and an amorphous nanogranular texture persisting in the outer shell. Electron energy-loss spectroscopy, which is sensitive to nanoscale elemental composition, showed a lower Ca/P ratio at the periphery of *Hyp* foci, consistent with a more amorphous mineral character, suggesting that OPN may play a role in delaying the amorphous-to-crystalline transition. These aspects of nanoscale mineral maturation in mutant mice having elevated OPN implicate this protein as a fine-tuning regulator of mineralization kinetics, mineral composition, and mechanical properties of bone.

## Abstract 2

Using advanced microscopy methods, we show the effects of upregulated osteopontin on the microscale morphogenesis and potential crystallinity of ripening mineral foci during bone mineralization, as a precursor to crossfibrillar mineral tessellation.

## Graphical abstract

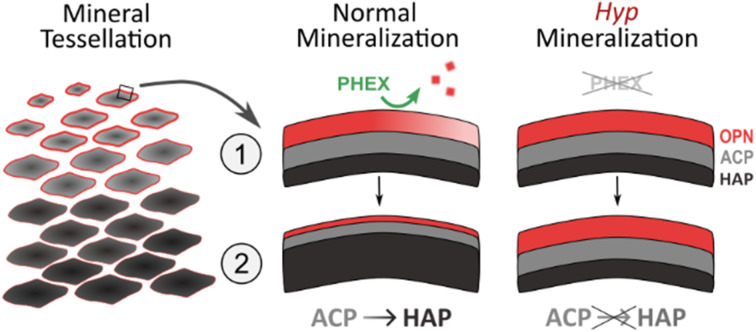

## Introduction

During lamellar bone formation, planar arrays of polarized osteoblasts lining bone surfaces unidirectionally secrete an organic, highly hydrated extracellular matrix called osteoid, which is rich in fibrillar type I collagen, noncollagenous proteins, small proteoglycans, and other small biomolecules. At this point, the assembled matrix matures through collagen and noncollagenous protein crosslinking,^1–4^ and other various protein interactions/modifications, and soon becomes primed for mineralization. The osteoblast cell layer permits ion passage from the bloodstream to the surface of the mineralizing bone, with the fluid in the mineralizing compartment being supersaturated in calcium and phosphate ions (with respect to the apatitic mineral phase of bone).^5^ It is now widely accepted that mineralization in bone and other mineralized tissues is regulated both systemically by mineral ion homeostasis^6^ and locally by small molecules and proteins.^7,8^ The calcium homeostatic system targets not so much the total body calcium content, but rather the concentration of calcium in the extracellular fluid. In a healthy individual, this value is remarkably stable over time, never deviating by >2% from its set point.^9^ The pathway from mineral ion supersaturation to mature mineralized tissue is dependent upon, among other factors, the important role played by mineralization inhibitors (such as pyrophosphate, PPi^10^). As opposed to the default inhibitory state that prevents ‘soft’ tissues from mineralizing, the removal of these inhibitors within forming bone by local, osteoblast-lineage cell expression of enzymes (such as tissue-nonspecific alkaline phosphatase, TNAP/ALPL, degrades inhibitory PPi) allows mineralization to proceed in ‘hard’ tissues – a notion we refer to as the Stenciling Principle for extracellular matrix mineralization.^11^

A more refined regulation of mineralization occurs through enzymatic removal of phosphoprotein inhibitors, such as osteopontin (OPN), by phosphate-regulating endopeptidase homolog X-linked (PHEX)^12^ in addition to the driving forces of classical ion supersaturation. When removal of mineralization inhibitors takes place in the bone (a driving force for mineralization to proceed), heterogeneous nucleation^13,14^ of nanoscopic mineral foci occurs within the extracellular matrix. In lamellar bone, the formation of these nascent foci breaks the relatively featureless ‘background’ of organic extracellular matrix *via* stochastic shifts in the metastable equilibrium of antagonistic mineralization inhibitors and promoters (inhibitors of inhibitors). This foci heteronucleation event is an example of spontaneous symmetry breaking, in that it occurs when the system develops instabilities that disrupt an initial symmetrical arrangement to then form diverse patterns and structures.^15^ Biological equilibria are often dynamic or metastable, where breaking of a featureless symmetry is based on stochastic fluctuations of the system.

The incipient mineral foci (<500 nm), having a quasi-periodic spacing in the osteoid near the mineralization front, rapidly grow over time through a 3D Voronoi-like process (nucleation, outward growth, and eventual impingement) to form a volume-filling tessellation pattern (with each repeating unit occupying roughly 1–2 μm^3^) within collagen fibril bundles and bone lamellae.^16^ These mature mineral elements are referred to as tesselles, having a final geometry constrained by the initial distribution of nucleated foci and the growth kinetics of the system. Tesselles are outlined by discrete organic- and water-rich boundaries and, in lamellar bone, have a final geometry roughly approximated by a prolate ellipsoid.^17^ The rates at which new mineral foci first nucleate and then grow define this space-filling organization of the tessellation pattern observed in bone. The phenomenon of mineral nucleation is governed by factors that are not well-understood but presumably require local supersaturation of ionic calcium and phosphate, and modifications of hydration state. Before reaching a comparatively stable polycrystalline polymorph of carbonated apatite,^18,19^ bone mineral undergoes ripening through transient phases. This mineralization process is thought to be influenced by small biomolecules like pyrophosphate and by various organic matrix components such as the SIBLING family of phosphoproteins.^20^ Growth of nucleated foci consequently also requires a steady supply of calcium and phosphate ions originating from the circulation. This directional flow of mineral ions towards bone provides a more-or-less confluent equilibrium between circulating mineral ion concentrations and those of the bone tissue fluid.

The intrinsic disorder of OPN (classified as an IDP, an intrinsically disordered protein), and its demonstrated binding to various mineral phases (such as apatite and calcium carbonate, and to their transient amorphous mineral phases^21^) seems to be the most influential feature of this phosphoprotein in terms of its inhibitory potential. Select IDPs regulate mineralization *via* interactions with ions, ion clusters, and amorphous and crystalline phases of mineral. An open structure gives an IDP the ability to bind to more than one partner and present different surfaces to facilitate the regulation of mineralization. An open structure also facilitates exposure of post-translational modifications such as phosphorylation (extensive in bone OPN) – altering IDP net charge to provide greater structural and functional variability that opens the door to new ligands and binding sites, and potentially enables the IDP to more easily stabilize transient amorphous precursors as an inhibitor. It is likely that different combinations of each of the above exist *in situ*. We previously showed^22^ that inhibitors can be resistant to enzymatic degradation when bound to mineral, a finding that suggests the post-nucleation regulation of mineralization (in this case release from inhibition once nucleated) may be slowed in some cases, as may be the case for OPN binding to mineral. It is further shown in many studies by different groups including ours that, for ectopic calcification of collagen matrices and soft tissues, OPN is a reactionary inhibitor of unwanted calcification. OPN is also highly upregulated by tissue-resident cells (*i.e.*, not osteoblasts) that do not normally secrete abundant OPN, but do so only after ectopic mineral starts to precipitate in the collagen and/or elastin.^23^ A good example of this is in the extracellular matrix (elastin and collagen) of calcifying blood vessels, where smooth muscle cells secrete OPN upon calcification of the vessel wall (medial calcification, in humans, and in the MGP-knockout mouse^24^).

As an example of how a systemically circulating protein can influence mineralization, the plasma protein Fetuin-A produced by the liver has been suggested to recruit/bind calcium at sites of bone mineralization, stabilize pre-nucleation calcium–phosphate clusters, and form calciprotein particles attributable to the protein's numerous negatively charged aspartic acid and glutamic acid residues.^25,26^ Akin to Fetuin-A, a highly phosphorylated and intrinsically disordered protein that also strongly binds to mineral phases on account of its Asp-rich, Glu-rich, and phosphorylated Ser residues^27^ is OPN, a prominent inhibitory protein ^28^ found in and around mineralized interfaces.^29^ OPN is found in essentially all mineral deposits in mammals, but not in tooth enamel, whether the mineralization is physiologic or pathologic. With biological mineralization in bone occurring through such a clear example of symmetry breaking, we aim to decipher the role OPN plays in modulating the transition from mineral foci to mineral tesselles and to identify the pathway that patterns bone mineral at the microscale.

Mineral ion homeostasis in healthy individuals is regulated in part by fibroblast growth factor 23 (FGF23). Excessive FGF23 has been identified as a pivotal phosphaturic factor leading to renal phosphate wasting and the subsequent development of rickets and osteomalacia. FGF23 is a small circulating hormone, primarily secreted by osteocytes, that balances mineral ion homeostasis by acting upon regulatory mechanisms in the kidney that control renal phosphate excretion.^30^ FGF23 also controls OPN levels.^31^ It is implicated in the pathogenesis of various renal phosphate-wasting diseases, including X-linked hypophosphatemia (XLH), autosomal dominant hypophosphatemic rickets (ADHR), oncogenic osteomalacia (OOM), chronic kidney disease (CKD), familial tumoral calcinosis (FTC), McCune–Albright syndrome, and fibrous dysplasia of bone.

The *Hyp* mouse, a phenocopy model for X-linked hypophosphatemia (XLH), exhibits a phenotype characterized by hypophosphatemia due to impaired renal tubular reabsorption of phosphate, like in XLH. This leads to rickets/osteomalacia, marked by defective bone mineralization (hypomineralization). The *Hyp* mouse harbors a large deletion in the PHEX gene^32^ and elevated levels of FGF23 are also observed in these mice, contributing to the phosphate-wasting phenotype.^33^ Additionally, the *Hyp* mouse displays impaired vitamin D metabolism that alters mineral ion homeostasis.^33^

In contrast, loss of the FGF23 hormone in the *Fgf23*-knockout mouse mice leads to high serum phosphate, calcium, and 1,25-vitamin D levels, resulting in early mortality attributable to severe ectopic soft-tissue calcifications and organ failure. FGF23-deficient mice exhibit a phenotype that includes profound growth retardation, muscle wasting, infertility, atherosclerosis, extensive soft-tissue calcifications, pulmonary emphysema, general tissue atrophy, severely shortened life span, and biochemical disorders including hyperphosphatemia, hypercalcemia, high serum 1,25(OH)_2_D levels, and decreased serum parathyroid hormone (PTH) levels.^34–36^ Despite the presence of such a high serum mineral ion content and even the presence of severe soft-tissue calcifications, Fgf23-knockout mice present with severe hypomineralization defects in skeletal mineralization. The reason for this reduced skeletal mineralization occurring in the presence of high serum calcium and phosphate suggests the accumulation of a mineralization inhibitor locally within the extracellular matrix. Conversely, recent studies^37,38^ have demonstrated that overexpression of FGF23 in mice leads to hypophosphatemia and hyperphosphaturia.

In this study, we have focused on the pathway of incipient bone mineralization near the mineralization front with an emphasis on mineral foci abundance and characterization, and on the role that OPN might play in locally regulating nanoscale mineral foci growth and microscale mineral tessellation. We do this primarily by using electron microscopy and characterization methods for normal (wild type, WT) mouse bone and for well-established mutant mouse models that phenocopy human osteomalacic diseases in having defective mineralization (hypomineralization), altered mineral ion homeostasis, and elevated inhibitory OPN. These mutants include a mouse model of X-linked hypophosphatemia (the *Hyp* mouse – a spontaneous mutation inactivating the PHEX enzyme) and a transgenic mouse model where the fibroblast growth factor 23 (FGF23) gene has been deleted (the *Fgf23*^−/−^ mouse). Both mouse strains display severe osteomalacia and provide models where one has a moderate increase in OPN (the *Hyp* mouse), and the other has a very high level of OPN (the *Fgf23*^−/−^ mouse), thus providing a graded context for our conclusions on the function of OPN in the nanoscale mineralization pathway.

## Results and discussion

### The mineralization pathway of bone and its relationship to osteopontin

**Fig. 1:**
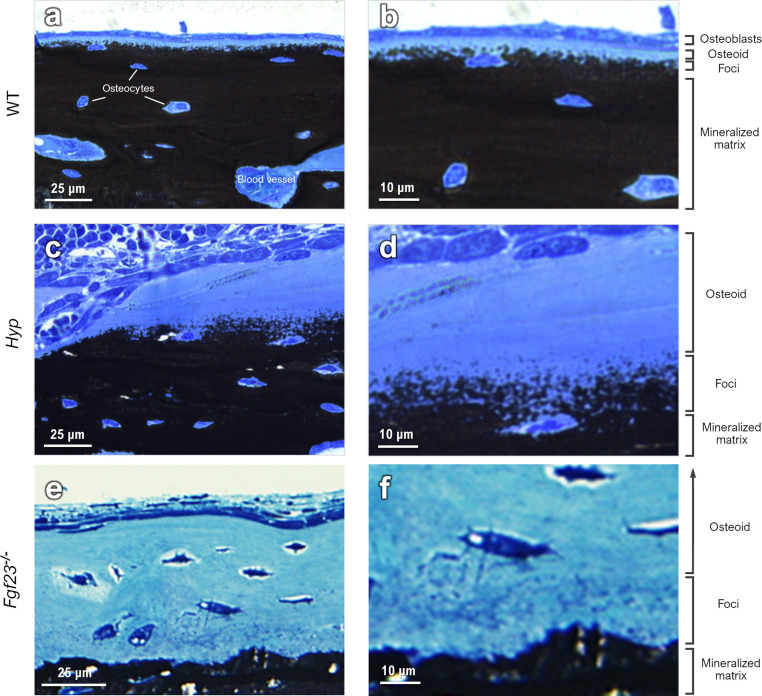
Light microscopy of the mineralization front in long bones from the limbs of WT, *Hyp* and Fgf23^−/−^ mice. (a and b) In WT bone, formation of mineral foci occurs close to the bulk mineral starting at a clearly defined mineralization front at the interface between the thin osteoid and the mineralized bone proper. (c and d) In *Hyp* mice, there is roughly a 6-fold increase in osteoid thickness and mineral foci are more diffusely distributed across a larger volume of bone matrix that extends outwards from the bulk of the mineralized bone tissue. (e and f) In *Fgf23*^−/−^mice having normal-to-high serum calcium and phosphate levels, and a 2-fold and 13-fold increase in bone OPN and in serum OPN, respectively, the thickness of the osteoid layer increases to an even greater extent than in *Hyp* mouse bone, and also presents the characteristic texture of mineral foci.

To draw a link between nanoscale-to-microscale macromolecular interactions occurring in normal lamellar bone, and how they deviate in osteomalacic bone to result in the clinical symptoms of diseases such as X-linked hypophosphatemia, one must consider the kinetics of lamellar bone apposition. With healthy nutritional status, forming bone has an osteoid thickness of 5–7 μm (the distance between the surface osteoblast layer and the mineralization front) depending on the species, anatomical location, age, and physical activity (Fig. 1a and b), and a mineral apposition rate (MAR) of approximately 1 μm per day.^39^ In histomorphometric terms, osteomalacia is defined as the simultaneous occurrence of increased osteoid thickness and increased mineralization lag time.^40–42^ In WT mice, there are notably fewer discrete mineral foci, and they are only located in close proximity to the mineralization front – as they grow and pack into the bulk mineral compartment (mineralized bone proper). In defective mineralization (hypomineralization, osteomalacia) such as in the long bones of *Hyp* mice, a reduced MAR results in severe osteoidosis, where the osteoid layer can increase in thickness by up to approximately an order of magnitude (Fig. 1c and d). Since the mineralization front in *Hyp* mice does not have a clear and continuous boundary, exact comparative measurements of osteoid thickness cannot be readily made. Bone mineral foci in the *Hyp* mouse are highly abundant in the osteoid near what might be considered a diffuse mineralization front, and they have a characteristic distribution outwards from the bulk of the mineralized bone tissue (Fig. 1c and d). Forming lamellar bone in *Fgf23*^−/−^ mice have both a dramatically increased osteoid thickness and an abundance of isolated mineral foci, occupying a deeper layer of the osteoid (an extra 2–3 μm; Fig. 1e and f).

**Fig. 2:**
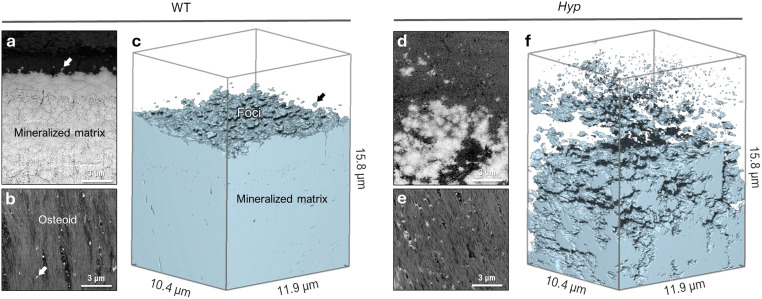
Microscale texture of mineral in mineralizing WT and *Hyp* mouse tibial bone. (a) Backscattered electron (BSE) SEM image showing the typical appearance of the mineralization front and lamellar bone organization in cross-section in a WT mouse. Nascent mineral foci (arrow) only nucleate close to the bulk mineral. (b) Planar tangential section through the osteoid of mineralizing WT mouse bone, showing sparse nucleation of mineral foci (arrow) amongst the type I collagen fibril bundles. (c) 3D FIB-SEM reconstruction of thresholded bulk mineral in a volume of WT bone tissue. (d) In *Hyp* mouse bone, a BSE-SEM cross-section image showing the presence of a large population of irregular and erratic mineralization foci near the diffuse region of a ‘mineralization front’. (e) Planar tangential section through the osteoid of mineralizing *Hyp* bone, showing the distribution of numerous mineral foci in bundled collagen fibrils. (f) In *Hyp* mouse bone, 3D FIB-SEM reconstruction of abundant mineral foci of variable size, and an arrested/delayed mineral tessellation pattern (giving a four-fold increase in mineral surface area).

3D focused ion beam–scanning electron microscopy (FIB-SEM) operating in serial-surface-view (Slice & View) mode generates high-resolution imaging datasets comprising hundreds to thousands of sequential images that, when reconstructed, provide quantifiable volumes of the mineral distribution in bone. This allows for qualitative imaging and quantitative measurement of mineral patterning in normal and osteomalacic bone as it starts/develops in the osteoid and then extends across the mineralization front into the mineralized bone proper (Fig. 2). Voxel-wise measurement of the mineral surface area-to-volume ratio (each generated using the same voxel and volume size) in WT and *Hyp* bone yields a value of 1.84 μm^2^ μm^−3^ in WT bone and a much greater value of 8.05 μm^2^ μm^−3^ in *Hyp* bone. The reduced size of mineral foci together with an incomplete mineral tessellation, results in a 4-fold increase in specific surface area in the mineralizing *Hyp* bone – where foci are putatively stabilized by the excessive OPN (secreted by nearby osteoblasts and osteocytes). Electrostatic binding to calcium by highly phosphorylated and Asp- and Glu-rich OPN^43^ inhibits the growth of established mineral foci while not preventing the formation of nascent foci, thereby halting/delaying the foci-to-tesselle transition. Secreted OPN present in the osteoid of *Hyp* bone persists in the matrix since it is not enzymatically degraded (due to of the absence of PHEX activity in the *Hyp* mice) and binds to the 4-fold higher mineral surface area. Undegraded inhibitory OPN thus accumulates in the *Hyp* bone extracellular matrix, which acts together with low serum phosphate to continually ‘frustrate’ the growth of mineral foci.

Whereas *Hyp* mice are essentially a complete knockout of *Phex*, human patients with inactivating mutations in *PHEX* continue to have some, albeit limited, degree of OPN degradation, whose variances contribute to the degree of severity of the disease in XLH patients. As PHEX degradation of OPN proceeds (at whatever level) to release its inhibitory regulation in XLH patients, the continuum of growth of mineral foci towards mineral tessellation slowly continues at these sites. In *Hyp* mice with no PHEX activity, the phenotype is severe, with low serum phosphate and high levels of undegraded accumulating inhibitory OPN^12^ (along with increased gene expression of *Opn*/*Spp1*,^44^ causing massive osteomalacia and odontomalacia).

**Fig. 3:**
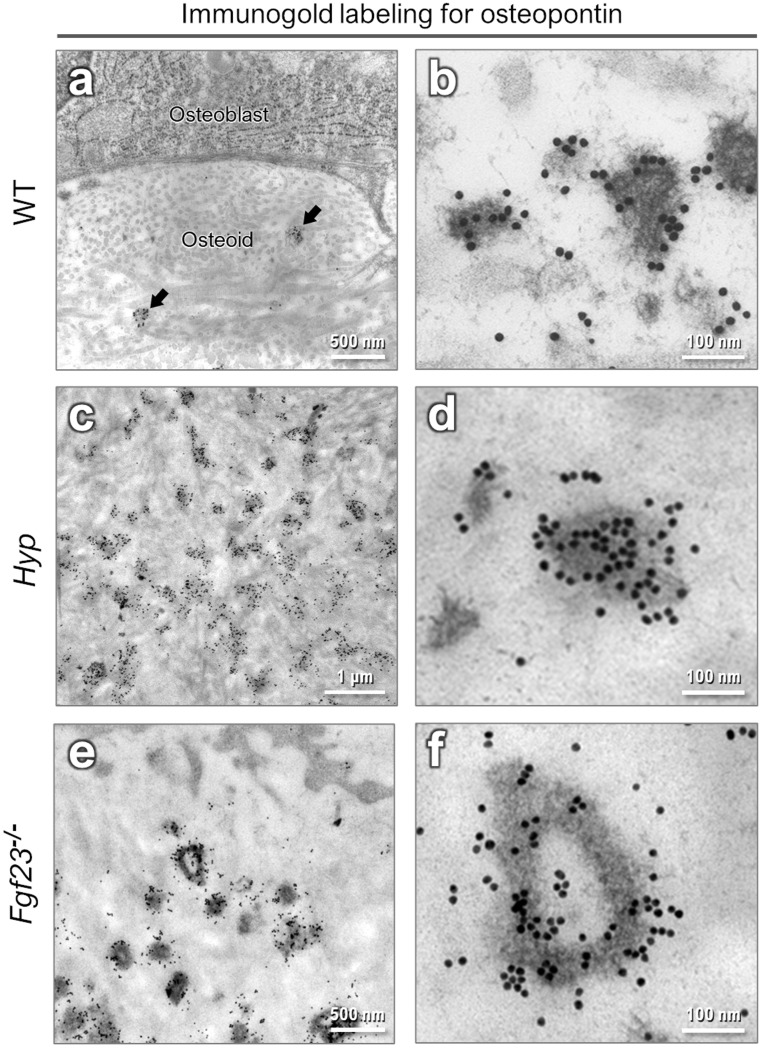
Immunogold labeling for osteopontin related to mineral foci in mouse limb bone. (a and b) In WT mice, mineral foci (arrows) containing OPN are relatively sparse in the osteoid. Little or no OPN labeling is observed elsewhere in the osteoid. (c–f) Mineral foci with strong immunogold labeling for OPN are far more abundant in the increased osteoid of *Hyp* and *Fgf23*^−/−^ mice.

Here we show that focus growth is substantially altered depending on OPN levels in the local microenvironment. Transmission electron microscopy of immunogold-labeled (for OPN) thin sections of forming bone corroborate OPN's inhibitory role in the foci-to-tessellation transition. In conditions where OPN is locally elevated (XLH/*Hyp* PHEX deficiency, and FGF23 deficiency) and at normal levels (WT), immunogold labeling co-localized this mineral-binding inhibitory protein to nascent mineral foci (Fig. 3) as a post-nucleation event. In the osteoid, immunogold labeling for OPN was predominantly associated with the mineral foci.

**Fig. 4:**
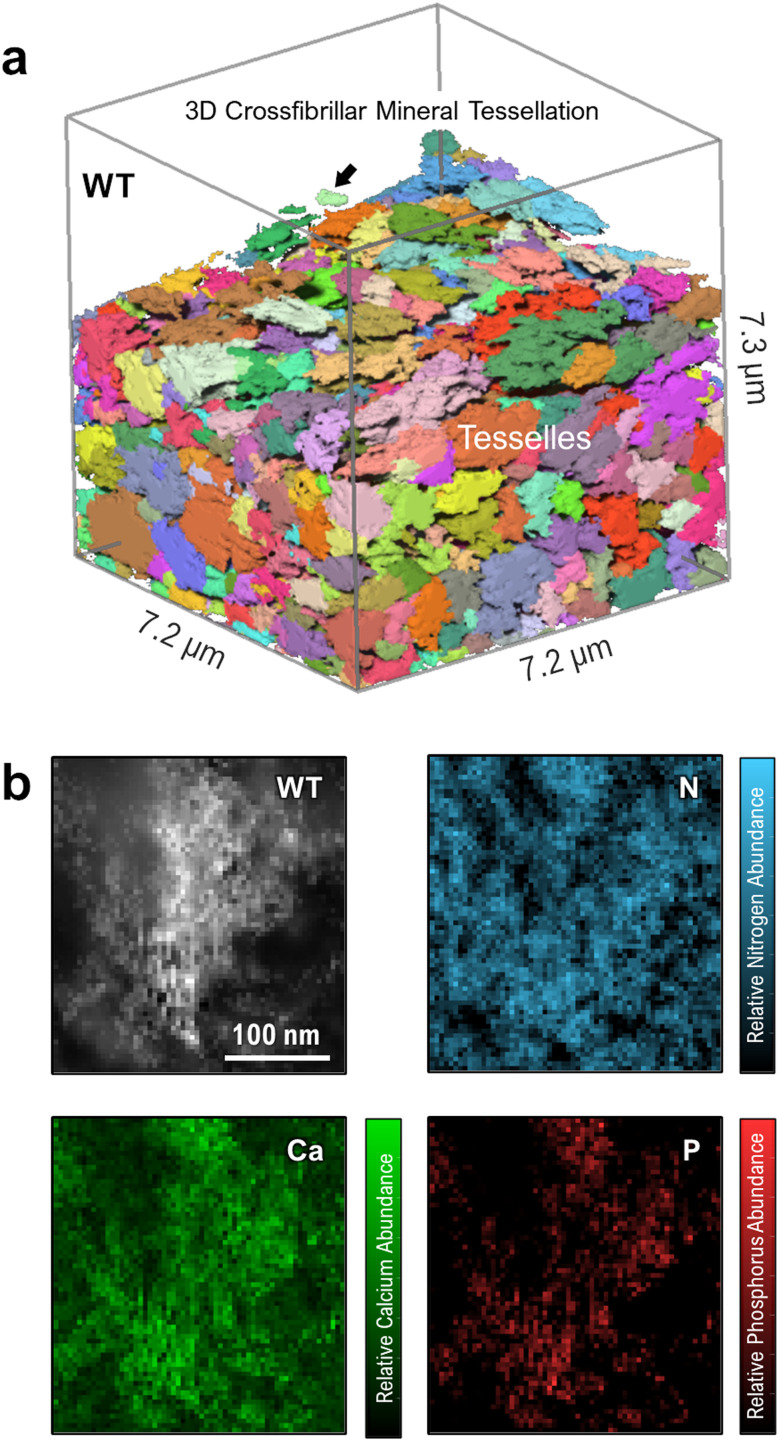
Structure and composition of WT mouse tibia mineral tessellation. (a) FIB-SEM reconstruction of a normal mineral tessellation pattern. A relatively large mineral focus is shown by the arrow. Unmineralized osteoid has been digitally removed to highlight only the mineral phase. (b) High-angle annular dark-field scanning transmission electron microscopy (HAADF-STEM) image and EELS elemental composition maps of N, Ca and P for a large mineral focus near the mineralization front.

Until now, few works have described how elevated OPN levels affect calcium-phosphate mineral polymorphism and elemental atomic ratios in newly formed mineral foci. This study characterizes the incipient mineral foci and their growth to larger mineral foci prior to tessellation, using electron energy-loss spectroscopy (EELS). Fig. 4 shows a WT mouse lamellar bone volume with mineral tessellation as obtained *via* FIB-SEM tomography (Fig. 4a), and an EELS chemical elemental distribution map for a large mineral focus near the mineralization front (Fig. 4b). The elemental maps indicate the relative abundance of calcium, phosphorus and nitrogen, the latter deriving from organic material.

### The role of osteopontin in shaping mineral polymorphism

**Fig. 5:**
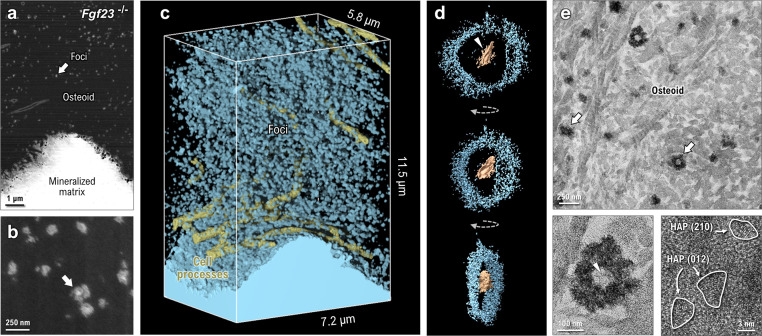
Core–shell mineral focus morphology in FGF23-deficient bone mineralization. (a) A single 2D slice from FIB-SEM imaging showing the high abundance of small mineral foci common in cases of elevated OPN. (b) Higher-magnification SEM image showing foci with a bright mineral interior and a surrounding diffuse mineral shell structure. (c) Segmentation of the 3D FIB-SEM dataset showing the 3D distribution of mineral foci and cell processes within the osteoid. The high surface area-to-volume ratio is reminiscent of *Hyp* mouse bone mineralization, resulting in a slower progression towards a tessellated mineral pattern. (d) Electron tomography reconstruction through the midsection of a single core–shell mineral focus. The core of the focus tends to be more platy, with a granular mineral populating the shell layer and showing little sign of platelet structure. (e) Bright-field TEM imaging of core–shell structures. Mineral foci without a clear core could be caused by core elements positioned out of the plane-of-section or a direct mineralization of collagen fibrils. Platy mineral can be observed in the central core of the mineral foci (arrowhead in the bottom left panel of e) whereas the peripheral shell is more amorphous in nature, with some small crystalline nanodomains (outlined in the lower right panel of e). Images in panel e were from sections having additional uranyl acetate and lead citrate counterstaining. In all panels of this figure, arrows indicate mineral foci, and arrowheads indicate platy mineral in the core of the foci.

Focused ion beam–scanning electron microscopy in the OPN-enriched mineralizing bone of the FGF23-deficient mouse reveals an abundance of newly formed mineral foci dispersed amongst cell processes within the osteoid (Fig. 5a). The majority of these foci, which have a diameter of approximately 150 nm and are composed of calcium-phosphate mineral, take on a globular appearance at higher resolution with no preferential elongation in any direction. When digitally sectioned through their midplane, these very small and uniform foci appear to have a bright core of calcium-phosphate mineral surrounded by a more diffuse ring-like structure also containing calcium and phosphate (Fig. 5b). Although these foci are small – as expected from an arrested growth state presumably caused by the elevated OPN in these mice – FIB-SEM reconstruction of the mineralized matrix shows that they densely populate the osteoid with an abundance and spacing consistent with a symmetry-breaking heteronucleation (Fig. 5c).

The core–shell form of the mineral foci found in FGF23-deficient mice can be discriminated and imaged in 3D using electron tomography (Fig. 5d). Reconstructions of the imaging volume show that the central region of the foci (bright in SEM imaging, dark in bright-field TEM) contains compartmentalized mineral in the form of single or multiple platelets (arrowheads in Fig. 5d and e), while the diffuse peripheral ‘halo’ of the foci is composed of irregularly shaped nanogranules with no long-range crystalline order (similar in morphology to nanogranular mineral described by others^45–47^). As shown in 2D micrographs of the osteoid (Fig. 5e), the largest foci seem to have a clear core–shell segregation of the mineral phase. At a glance, smaller mineral foci appear to solely contain granular shell-type mineral (with a few plate-like inclusions), but this is likely attributable to the level of the sectioning plane, where the core of the structure can lie outside of the thin tissue section required for TEM. High-resolution TEM of the outermost, nanogranular shell of the mineral shows the presence of a poorly crystalline mineral phase, with only small crystalline domains having visible lattice fringes. The sparse crystalline regions of the granular ‘shell’ are roughly 5 nm in diameter, where the *d*-spacings across these three crystallites are 3.20 Å, 3.18 Å, and 3.11 Å (potentially corresponding to the (012), (012), and (210) planes of hydroxyapatite, respectively).

Although core–shell segregation (centrally crystalline and peripherally more amorphous) of mineral foci is seemingly ubiquitous in the FGF23-deficient condition, this is not necessarily the sole form of mineralization occurring in the bones of these mice. Indeed, the irregular appearance of the mineralization front (core–shell foci in the osteoid as opposed to densely mineralized bulk tissue) in these mice hints at a diversity of mineralization mechanisms. Sparse core–shell mineral structures can be observed in a variety of mineralized tissues including calcified cartilage (manuscript in preparation), but in FGF23-deficiency there is a preponderance of core–shell structures compared to other 3D volumes of pathological and healthy bone tissue (where direct nucleation of crystallites in and around collagen fibrils is more dominant). The core–shell morphology may be attributable to the abundance of inhibitory OPN in the case of FGF23-deficiency. The core crystallization may be made possible by local depletion of OPN in the local milieu, enveloped by a peripheral amorphous ‘shell’ stabilized by OPN (like what has been described by Goobes and co-workers^48^).

The crystallization process in biominerals can follow nonclassical pathways, often forming intermediate phases with low degrees of crystallinity.^49^ This has been best documented for calcium-carbonate and calcium-phosphate mineralization systems. Bone mineralization is known to be one such case that follows this multi-pathway route, where amorphous and poorly crystalline precursors of apatite have been seen in the mineralizing fins of zebrafish in the form of similar small nanoparticles.^50^ Direct nucleation of apatite crystallites around, between, and within the type I collagen fibrils in bone (as recently visualized by Reznikov *et al.*^51^) is a longstanding and widely accepted phenomenon in the field of bone mineralization, presumably occurring in tandem with core–shell nucleation events. While information on promoters of *in vivo* bone mineralization is scant (see for example ref. 52 and 53), we recognize that the mineral ion concentrations in the extracellular matrix tissue fluid are in a metastable supersaturated equilibrium with respect to the mineral phase. Following the symmetry-breaking event of heterogenous mineral nucleation, the local release from inhibition across the background field of osteoid extracellular matrix becomes especially important. This is our current working model of how growth of heterogenous mineral foci can occur in bone – a stochastic release from a metastable inhibition. This view does not preclude there being primed mineralization sites chemically ‘imprinted’ onto or within the collagen fibrils, or between the collagen fibrils to provide molecular-level specific nucleation sites for mineral. For example, recent studies by the M. Duer group have shown Ca-containing liquid nanodroplets at specific sites along the collagen fibril surface that contain abundant poly(ADP ribose) (PAR), produced through the actions of PAR polymerase enzyme 1 (PARP1).^54^ More generally, following stochastic symmetry breaking to induce mineral nucleation at specific sites after local release from inhibition (perhaps achieved by TNAP enzyme degrading inhibitory pyrophosphate), we envisage that OPN then stabilizes post-nucleation mineral foci to carefully regulate their growth pathway towards microscale tessellation.

Mineral foci in the Fgf23^−/−^ mouse (Fig. 5) are far more numerous than those in WT mice. Here, the symmetry-breaking nucleation of foci can proceed unimpeded (mainly driven by ion supersaturation and extracellular matrix composition), but the abundance of foci is not necessarily indicative of an increased nucleation rate. The delay in focus growth and corresponding preservation of unmineralized matrix (by mineralization inhibitors) instead provides additional time for foci nucleation to occur, leading to more abundant nuclei.

Nanoscale spectroscopy in TEM, coupled with the cryo-preservation of bone used here (high-pressure frozen, vitreous ice), offers a means to characterize this early focus mineral through an instantaneous ‘snapshot’ in time and space that might reveal an amorphous-to-crystalline transition in its native hydrated environment. This cryogenic specimen preparation method^55^ in combination with freeze-substitution (both used here) provides a greater likelihood of preserving labile amorphous phases for observation and characterization in their native hydrated state, avoiding conventional solvent dehydration that may artifactually lead to crystallization events as water is depleted from the sample. The conventional dehydration and embedding approaches can be successfully applied when routinely examining other features of bone tissue that do not require a fine structural analysis or examination of small amounts of precursor phases.

**Fig. 6:**
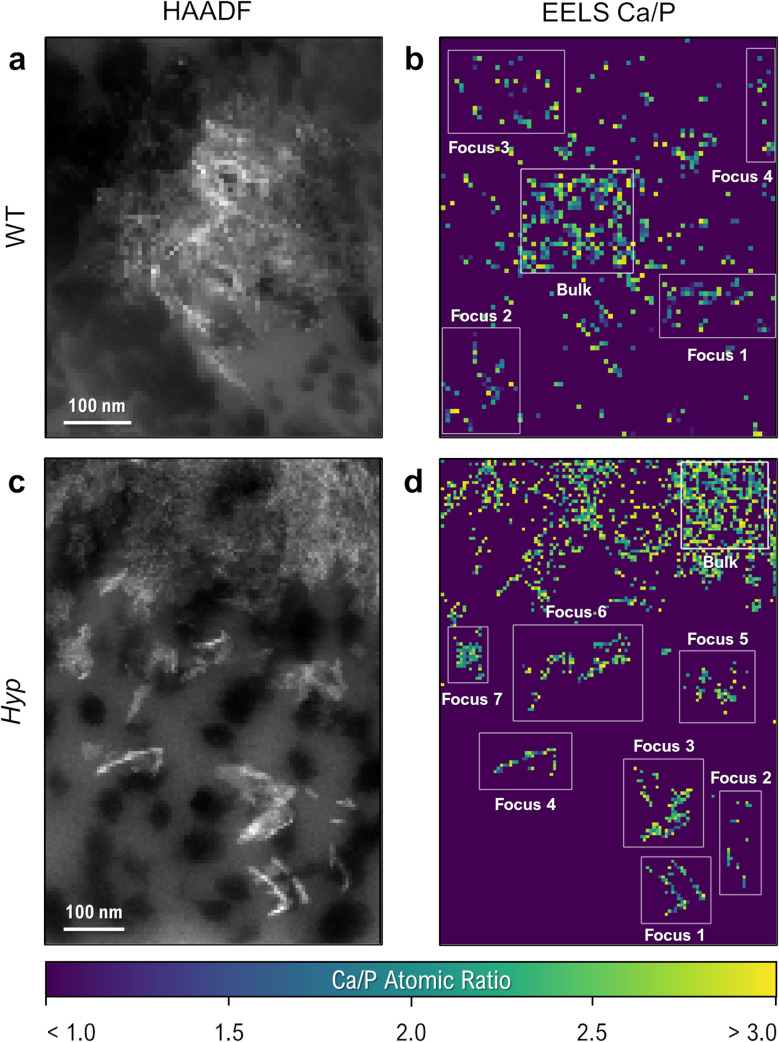
HAADF images and EELS Ca/P ratio maps of mineralization foci in (a and b) WT and (c and d) *Hyp* mice. In WT mouse lamellar bone, besides the more obvious, large established focus (labeled bulk), small clusters of calcium- and phosphorus-containing pixels in the surrounding tissue (labeled as Foci 1–4) are suggestive of incipient mineralization foci.

One developing focus at the mineralization front of a cryo-preserved WT tibia is shown in the HAADF image of Fig. 6a, where the focus itself is composed of smaller mineral crystallites. Individual cross-sectioned collagen fibrils (dark circular areas, 30–60 nm in diameter) can be seen in the unmineralized extracellular matrix surrounding the focus. Using an EELS spectral image map taken from the same region, the constituent composition maps of calcium and phosphorus can be used to generate an approximate calcium-to-phosphorus ratio for each pixel in the spectral image (Fig. 6b). In an analogous region of lamellar bone in the *Hyp* mouse, nanoscale-sized and OPN-rich mineral foci are more abundant (Fig. 6c and d), with their distribution and size matching the 3D reconstruction from FIB-SEM imaging.

With each pixel in the spectral image representing an area of less than 50 nm^2^, the maps provide a highly localized measurement of the Ca/P ratio (sampling roughly 2000 atoms within a single pixel). Given some sensitivity of N and O spectra to electron dose during spectral image acquisition (ESI†), these Ca/P ratios provide a robust and thickness-agnostic means of assessing local biomineral composition. In WT bone, despite the lack of clear platelet morphology in some regions of the ‘unmineralized’ matrix, small clusters of pixels are present that contain values within the broad range of calcium-phosphate mineral polymorphs (on the order of 1.0–3.0 Ca/P). Defining these (and any other small clusters of calcium phosphate) as early incipient mineral foci, comparisons of mineral composition can be made within a single spectral image by comparing the Ca/P ratios of the small foci to those of the ‘bulk’, more mature mineral at the core of a larger mineral focus or tesselle.

Table 1 shows the Ca/P ratios obtained using EELS for the depicted WT and *Hyp* bone mineral foci depicted in Fig. 6, expressed as relative percentages of a mineral-dense region within each image. In both cases, the measured Ca/P ratios have high variance and are marginally higher than typical reported values for amorphous calcium phosphate (∼1.50)^56^ and stoichiometric hydroxyapatite (∼1.66),^57^ which reasonably can be attributed to noise in the spectral images and the corresponding consistency of background subtraction. However, relative comparisons within the same image (generated using the same background subtraction in the spectra) remain possible. Based on the reported Ca/P values, we can expect a reduction in Ca/P ratio on the order of 10% when examining an ACP polymorph.

**Table 1:** Atomic Ca/P ratios of established large mineral foci and incipient mineral foci in WT and *Hyp* mouse bone from EELS mapping. Pixel count indicates the number of pixels with 1 < Ca/P-ratio < 3. Ratios for incipient/small foci are expressed as a percentage of the Ca/P ratio in a mature large focus (bulk). Incipient foci in WT mouse bone are observed to have a lower Ca/P ratio than the large established focus, this being consistent with the canonical notion of crystal ripening/maturation from amorphous precursors. Small foci in *Hyp* mice have the same Ca/P ratio as the mature large focus, suggesting a possible retardation of the amorphous-to-crystalline transition

	Region	Pixel count	Mean Ca/P ratio	Median Ca/P ratio
WT	**Bulk**	**218**	**−(2.098 ± 0.513)**	**−(2.060)**
	**All foci**	**152**	**91.7% (1.923 ± 0.561)**	**90.3% (1.862)**
	Focus 1	55	88.4% (1.855 ± 0.526)^a^	83.9% (1.729)
	Focus 2	44	84.2% (1.767 ± 0.595)^a^	80.8% (1.664)
	Focus 3	39	101.5% (2.130 ± 0.535)	105.8% (2.180)
	Focus 4	14	100.3% (2.104 ± 0.485)	99.6% (2.052)
*Hyp*	**Bulk**	**380**	**−(2.350 ± 0.385)**	**−(2.391)**
	**All foci**	**376**	**97.0% (2.280 ± 0.428)**	**97.8% (2.338)**
	Focus 1	50	87.4% (2.054 ± 0.485)^a^	87.9% (2.101)
	Focus 2	21	95.9% (2.255 ± 0.374)	94.6% (2.2363)
	Focus 3	85	101.3% (2.381 ± 0.406)	101.2% (2.421)
	Focus 4	34	94.8% (2.227 ± 0.520)^a^	92.3% (2.207)
	Focus 5	43	102.2% (2.402 ± 0.352)	99.5% (2.378)
	Focus 6	90	98.8% (2.322 ± 0.428)	99.2% (2.373)
	Focus 7	53	93.8% (2.205 ± 0.329)^a^	89.2% (2.132)

a *p* < 0.05 compared to the bulk material.

Within the single WT spectral image, incipient mineral foci appear to have a lower Ca/P ratio than the large established focus (1.923 *vs.* 2.098, respectively – an 8.3% difference in the Ca/P ratio of the incipient foci). Given that mineralization events can proceed *via* amorphous-to-crystalline pathways^49^ or by dissolution of amorphous phases and reprecipitation of apatite, it follows that these potentially incipient WT bone mineral foci display a more amorphous character than the well-developed WT bulk mineral based on the difference in their Ca/P ratios. Comparing Ca/P ratios in small *Hyp* foci to a large *Hyp* focus shows a greater uniformity in the Ca/P ratio (Ca/P = 2.280 in the small foci and Ca/P = 2.350 in the bulk – only a 3.0% difference in the incipient foci). This may mean that *Hyp* bone preserves an initial amorphous polymorph in the small foci that persists during maturation/growth as a result of the excess OPN in their localized extracellular matrix environment.

**Fig. 7:**
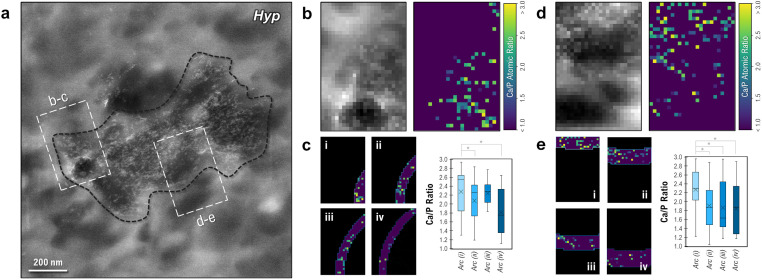
HAADF images and EELS Ca/P ratio maps at the edge of an isolated *Hyp* mineral focus. (a) Fine mineral crystallites (bright white) can be observed using HAADF throughout the bulk of the mineral focus (bounded by a black line), which in itself encapsulates a number of cross- (dark circular profile) or tangentially-sectioned (dark elliptical profile) individual collagen fibrils. (b and d) HAADF images and corresponding EELS maps of Ca/P ratios at the edge of the mineral focus (dashed white insets in a). Ca/P ratios roughly characteristic of bone mineral can be seen both inside and outside of the collagen profiles. (c and e) Partitioning each EELS Ca/P map into arc- or box-shaped subdomains/zones (i–iv) that run roughly parallel to the edge of the mineral focus (i = nearest to the center of the focus, iv = furthest from the center of the focus and at the focus external boundary) shows a statistically significant reduction (*p* < 0.05) in Ca/P ratio within a ∼120 nm layer at the mineral focus boundary. In the boxplots, ‘*X*’ denotes the mean value, box edges denote each quartile, and the centerline denotes the median. * Signifies *p* < 0.05.

To assess whether there is any gradient in amorphous character around a single mineral focus, low-dose EELS spectral images were acquired at the edges of an isolated, medium-sized cryogenically preserved *Hyp* focus (Fig. 7a). HAADF imaging (left panel of Fig. 7b) of the *Hyp* bone sample showed evidence of extrafibrillar mineral crystallites (white) surrounding a cross-sectioned collagen fibril (dark circular region at bottom of the left panel in Fig. 7b) in the region of interest. Mapping of Ca/P ratios using EELS (Fig. 7b right panel) showed a characteristic signal for calcium and phosphate in extrafibrillar mineral domains, with small clusters of mineral also located in the fibril itself. By partitioning this Ca/P map into zones of increasing distance from the focus midpoint (Fig. 7c), the relative degree of amorphous character was assessed using EELS at the growth front of a single *Hyp* focus. In the innermost zone (i, located closest to the center of the focus), a higher relative Ca/P ratio was observed (2.28) compared to those of sequential zones running closer to the actively mineralizing surface of the focus (2.07, 2.24 and 1.81, respectively). These findings were replicated in a second region of interest along this focus with zones running roughly parallel with the surface of the focus (Fig. 7d and e), where the relative Ca/P ratio successionally declined from 2.27 to 1.85 as the boundary was approached. Statistical significance was observed between the innermost rectangular band (i) and all other bands (ii, iii and iv) – suggesting that a minor decline in mineral crystallinity could be occurring toward the external boundary of the focus.

It is important to note that these EELS maps are highly dependent on the quality of the background subtraction for each particular spectral image (which becomes less reliable as electron dose decreases), and values should only be considered as relative comparisons to other values within the exact same image. It is also important to consider factors other than Ca/P ratio, which is only a proxy for the mineral crystallinity. With possible variation in organic phosphate abundance, such as phosphorylated serine amino acid residues abundant in OPN (although seemingly negligible for these analyses, as shown in the ESI†), and/or degree of lattice carbonation, the EELS spectral imaging in this work provides only a preliminary description of how spectral maps of relative atomic abundance can be used to supplement observations of polymorphic transitions or nanoscale chemistry changes in pathological and healthy mineralization systems, here studied in bone.

## Conclusions

Along with the systemic factors regulating mineral ion homeostasis, the local abundance of mineralization-regulating extracellular matrix proteins plays an important role in defining the chemistry, polymorphism, and kinetics of bone mineralization. These nanoscale protein–mineral interactions adaptively inhibit and refine mineral crystallization in normal bone, and by the same token define the severity of macroscale clinical symptoms in osteomalacia where OPN is upregulated or simply accumulates through lack of enzymatic processing. Besides the work presented here, numerous studies undertaken both *in vitro* and *in vivo* consistently point to OPN phosphoprotein as being a potent mineralization inhibitor often independent of changes in mineral ion homeostasis, whether constituently expressed in bone by osteoblasts/osteocytes or upregulated by tissue-resident cells in cases of ectopic calcification (*e.g*. ref. 24 and 58–61). Based on our observations, we summarize the following significant findings in bone pertaining to nanoscale mineral morphology based on our observations:

(i) In hypophosphatemic, osteomalacic *Hyp* (PHEX enzyme-deficient) mouse lamellar bone, hypomineralization of the bone is caused by renal phosphate wasting and by excessive OPN accumulated in the extracellular matrix that binds to and stabilizes small mineral foci formed in the osteoid, retarding and altering mineral tessellation in the vicinity of the mineralization front.

(ii) Through a newly identified mineralization pathway observed in FGF23-deficient transgenic mice, where OPN accumulates in bone and is upregulated, nucleation of platy mineral (presumably crystalline) is confined to the center of core–shell mineral foci. The peripheral shell mineral phase of the foci is predominantly amorphous/poorly crystalline, appearing nanogranular in texture and putatively stabilized by the excessive OPN.

(iii) Low-dose EELS analysis of mineral foci in bone can detect minor changes in composition at the nanoscale, namely the Ca/P ratio, which can potentially be used to identify and interpret possible polymorphic transitions and crystallinity gradients around the periphery of mineral foci.

## Methods

### Animals, sample preparation, light and TEM microscopy, and immunogold labeling for OPN

#### *Hyp* and WT mice

Tibiae of newborn, and 3.5–5 months-old, normal male C57BL/6 wildtype (WT) and mutant *Hyp* mice (strain B6.Cg-*Phex*^*Hyp*^/J from the Jackson Laboratory, Bar Harbor, ME, USA) with truncation in the *Phex* gene were dissected and trimmed to isolate the diaphysis. Initial 24 h fixation with 2% paraformaldehyde in 0.1 M Na cacodylate buffer was completed for all samples. All samples were stained with Alcian blue for 4 h (pH 5.8) before a secondary overnight fixation in 4% glutaraldehyde. Chemicals and reagents were purchased from Thermo Fisher Scientific (Waltham, MA, USA) unless otherwise specified. After washing in 0.1 M Na cacodylate buffer, conductive staining was performed on all samples using repetitive exposure to osmium tetroxide (Electron Microscopy Sciences, Hatfield, PA, USA) and thiocarbohydrazide ligand (OTOTO method^62^). Samples were gradually dehydrated at room temperature to 100% acetone followed by infiltration and embedding in EPON epoxy resin (Electron Microscopy Sciences, Hatfield, PA, USA). These samples were used for light microscopy and FIB-SEM analyses. For light microscopy, 1-mm-thick survey sections were cut from the polymerized blocks on an ultramicrotome and stained for mineral using von Kossa reagent (3% silver nitrate) followed by counterstaining with toluidine blue.

Some bone samples were fixed in 4% paraformaldehyde plus 1.0% glutaraldehyde in 0.1 M sodium cacodylate buffer, pH 7.3 and decalcified for immunogold labeling in 8% EDTA over 2 weeks followed by embedding in LR White acrylic plastic (London Resin Company, Berkshire, UK). These had no osmication. For immunogold labeling of OPN before transmission electron microscopy, LR White 100 nm-thick sections were incubated with polyclonal goat anti-mouse OPN antibody (R&D Systems), followed by rabbit anti-goat secondary antibody (Sigma-Aldrich, St. Louis, MO, USA), and then protein A-colloidal gold (14 nm) conjugate (Dr G. Posthuma, University of Utrecht, The Netherlands).

Another set of newborn mouse bones were processed *via* cryo-fixation and cryo-embedding into EPON epoxy resin blocks. Bones from newborn mice were dissected and immediately frozen to vitreous ice using high-pressure freezing (Leica EM ICE) followed by freeze-substitution into EPON epoxy resin. These samples were used for light microscopy, (S)TEM and EELs analyses.

All animal procedures were reviewed and approved by Institutional Animal Care and Use Committees.

#### *Fgf23* ^−/−^ and WT mice

Bones from 3-week-old *Fgf23*^−/−^^31^ and WT littermate mice were embedded in plastic for histology *via* light and transmission electron microscopy and for high-resolution, immunogold ultrastructural labeling for OPN. Bones were fixed in 4% paraformaldehyde plus 1.0% glutaraldehyde in 0.1 M sodium cacodylate buffer, pH 7.3. Samples were left undecalcified for embedding in EPON epoxy resin (Cedarlane, Burlington, Canada). Samples destined for embedding in EPON for morphological analysis were additionally osmicated for 1 hour in potassium ferrocyanide-reduced 1% osmium tetroxide. Before embedding, all samples were dehydrated in either a graded acetone or ethanol series, infiltrated with the embedding media and placed into mounting molds, and the blocks were polymerized at 55 °C for 2 days. For light microscopy, 1-mm-thick survey sections were cut from the polymerized blocks on an ultramicrotome and stained for mineral using von Kossa reagent (3% silver nitrate) followed by counterstaining with toluidine blue. For transmission electron microscopy, 80 nm-thick sections were cut on the ultramicrotome followed by conventional staining with uranyl acetate and lead citrate, after which the sections were viewed in a FEI Tecnai 12 transmission electron microscope (Hillsboro, OR, USA) operating at 120 kV.

For some FGF23-deficient bone samples, mineral foci and mineralizing tissue were imaged *via* TEM using a Thermo Fisher Scientific Talos 200X transmission electron microscope at an accelerating voltage of 200 kV and using 100 nm sections taken from the tibial mid-diaphysis of a three-week-old mouse. Sections were stained with uranyl acetate and lead citrate before imaging. *D*-spacing was measured in ImageJ by taking the fast Fourier transform (FFT) of the high-resolution TEM image, thresholding a selection containing only the crystallographic plane of interest, taking the inverse FFT (iFFT) of this threshold, and sampling measurements on the plot profile of the iFFT.

Some bone samples were fixed in 4% paraformaldehyde plus 1.0% glutaraldehyde in 0.1 M sodium cacodylate buffer, pH 7.3, and decalcified for immunogold labeling in 8% EDTA over 2 weeks followed by embedding in LR White acrylic plastic (London Resin Company, Berkshire, UK). These samples had no osmication. For immunogold labeling of OPN before transmission electron microscopy, LR White 100-nm-thick sections were incubated with polyclonal goat anti-mouse OPN antibody (R&D Systems), followed by rabbit anti-goat secondary antibody (Sigma-Aldrich, St. Louis, MO, USA), and then protein A-colloidal gold (14 nm) conjugate (Dr G. Posthuma, University of Utrecht, The Netherlands).

### Focused ion beam–scanning electron microscopy (FIB-SEM) tomography

Bone samples from 4-month-old wildtype and *Hyp* mice were prepared as above. WT and *Hyp* blocks were trimmed to a volume of interest and sputtered with platinum prior to imaging. Focused ion beam–scanning electron microscopy (FIB-SEM) serial sectioning was conducted using a gallium liquid metal ion source on a Helios Nanolab 660 DualBeam microscope. The accelerating voltage and current were set to 30 kV and 0.79 nA, respectively, for the ion beam. Images were acquired using an electron accelerating voltage of 2 kV, probe current of 0.40 nA, pixel size of 17 nm, slice thickness of 17 nm, and dwell time of 30 μs.

FIB-SEM tomography was also conducted in mineralizing tissue of tibiae from a 3-week-old *Fgf23*^−/−^ mouse (as described in ref. 31). Following dissection, the bone was fixed in 4% paraformaldehyde + 1% glutaraldehyde in 0.1 M sodium cacodylate buffer, serially dehydrated in ethanol, and embedded in LR White acrylate resin. The block was trimmed to an appropriate region of interest and sputter-coated with 4 nm of platinum for electron microscopy. FIB-SEM was performed using a Thermo Scientific Helios 5 Hydra CX microscope using inductively coupled oxygen plasma as an ion source. Tomography acquisition was acquired in Auto Slice and View mode and using an ion accelerating voltage of 30 kV, ion current of 1.7 nA, slice thickness of 7 nm, electron accelerating voltage of 2 kV, electron current of 0.20 nA, 7 nm pixel size, dwell time of 4 μs (with twofold line integration and fourfold frame integration), and backscatter signal detected by the through-lens detector.

3D volumetric reconstructions were done in Dragonfly 3D World 2024.1. Segmentation of mineralized tissue in each of the three 3D volumes was done using grayscale thresholding.

### Electron tomography

Sections of Fgf23^−/−^ mouse femur from epoxy-embedded specimens were cut on an ultramicrotome at a thickness of 150 nm. Single-axis tomograms were collected using a Titan Krios TEM (Thermo Fischer Scientific), operated at 300 kV and equipped with a Falcon 2 DED (Thermo Fisher Scientific), using FEI Batch Tomography Software. The tilt series was collected at a magnification of 59 000 over a tilt range of ±60° with an angular increment of 2°. The nominal pixel size was 1.375 Å, the defocus ranged from −2 to 3 μm, and the total dose per tomogram was ∼80 e^−^ Å^−2^.

### Electron energy-loss spectroscopy (EELS)

Newborn mouse tibiae were processed *via* cryo-fixation and cryo-embedding into EPON epoxy resin blocks. Samples were dissected and immediately frozen to vitreous ice using high-pressure freezing (Leica EM ICE) and freeze-substitution embedding. These sample blocks were sectioned for light microscopy, from which regions of interest were identified and then thin-sectioned for TEM and EELS spectroscopy analysis.

High-angle annular dark-field scanning transmission electron microscopy (HAADF-STEM) and EELS spectral images were acquired using a Thermo Fisher Scientific Talos 200X transmission electron microscope using an accelerating voltage of 200 kV, dispersion of 0.3 eV per ch, probe size of 2 Å, and temperature of ∼150 K using a liquid-nitrogen-cooled Gatan holder (model 613). Power-law background subtractions were performed separately at the P L_2,3_-edge, Ca L_2,3_-edge, N K-Edge, and O K-edge for each spectral image using Digital Micrograph. Ca/P ratios were generated from the Ca and P elemental maps using the Simple Math tool in Digital Micrograph before importing into Dragonfly 3D World. Ca/P ratios less than 1.0 or greater than 3.0 were assigned a value of 0.0 to isolate pixels roughly corresponding to bone mineral. The distribution of Ca/P ratios for each focus were compared to the corresponding bulk region in the same image using the chi-square test, with significance set at *α* = 0.05.

Low-dose (∼30–75 e^−^ Å^−2^) HAADF images and EELS maps were acquired at the edge of an isolated mineral tesselle using a monochromated Nion HERMES 100 STEM operated at 60 kV with a dispersion of 0.5 eV per ch, dwell time of 1 s, and temperature of ∼110 K using a liquid nitrogen cooled ELSA Gatan holder. Spectral images were sampled across a 240 nm × 360 nm area using 10 nm × 10 nm pixels, and calcium-to-phosphorus ratios were mapped according to the aforementioned technique. Spectral images were separated into arc-shaped bands in Dragonfly according to their proximity to the edge of the tesselle. Statistical comparisons between each band in a single spectral image were calculated using a one-way ANOVA in R, using a significance level of *α* = 0.05. Ca/P ratios across multiple background subtractions are reported in Table S1† for a single EELS map, which can be used to estimate an approximate error range for the values.

## Conflicts of interest

All authors declare that they have no competing interests related to this work.

## Supplementary Material

FD-261-D5FD00013K-s001

## Data Availability

The data supporting this article have been included as part of the ESI.†

## References

[cit1] Knott L., Bailey A. J. (1998). Collagen cross-links in mineralizing tissues: a review of their chemistry, function, and clinical relevance. Bone.

[cit2] Kaartinen M. T., El-Maadawy S., Rasanen N. H., McKee M. D. (2002). Tissue transglutaminase and its substrates in bone. J. Bone Miner. Res..

[cit3] Christensen B. (2016). *et al.*, Transglutaminase 2-Catalyzed Intramolecular Cross-Linking of Osteopontin. Biochemistry.

[cit4] Christensen B. (2014). *et al.*, Identification of transglutaminase reactive residues in human osteopontin and their role in polymerization. PLoS One.

[cit5] Glimcher M. J. (2006). Bone: Nature of the Calcium Phosphate Crystals and Cellular, Structural, and Physical Chemical Mechanisms in Their Formation. Rev. Mineral. Geochem..

[cit6] Campbell A. K. (1990). Calcium as an intracellular regulator. Proc. Nutr. Soc..

[cit7] Boskey A. L. (1981). Current Concepts of the Physiology and Biochemistry of Calcification. Clin. Orthop. Relat. Res..

[cit8] Glimcher M. J. (1989). Mechanism of calcification: role of collagen fibrils and collagen-phosphoprotein complexes in vitro and in vivo. Anat. Rec..

[cit9] Houillier P., Froissart M., Maruani G., Blanchard A. (2006). What serum calcium can tell us and what it can't. Nephrol., Dial., Transplant..

[cit10] Murshed M., Harmey D., Millan J. L., McKee M. D., Karsenty G. (2005). Unique coexpression in osteoblasts of broadly expressed genes accounts for the spatial restriction of ECM mineralization to bone. Genes Dev..

[cit11] McKee M. D., Buss D. J., Reznikov N. (2022). Mineral tessellation in bone and the stenciling principle for extracellular matrix mineralization. J. Struct. Biol..

[cit12] Barros N. M. (2013). *et al.*, Proteolytic processing of osteopontin by PHEX and accumulation of osteopontin fragments in Hyp mouse bone, the murine model of X-linked hypophosphatemia. J. Bone Miner. Res..

[cit13] Fleisch H. (1964). Role of nucleation and inhibition in calcification. Clin. Orthop. Relat. Res..

[cit14] Glimcher M. J. (1959). Molecular Biology of Mineralized Tissues with Particular Reference to Bone. Rev. Mod. Phys..

[cit15] Ball P. , Shapes: Nature's Patterns: a Tapestry in Three Parts, Oxford University Press, 2011

[cit16] Buss D. J., Reznikov N., McKee M. D. (2020). Crossfibrillar mineral tessellation in normal and Hyp mouse bone as revealed by 3D FIB-SEM microscopy. J. Struct. Biol..

[cit17] Binkley D. M., Deering J., Yuan H., Gourrier A., Grandfield K. (2020). Ellipsoidal mesoscale mineralization pattern in human cortical bone revealed in 3D by plasma focused ion beam serial sectioning. J. Struct. Biol..

[cit18] Rey C., Combes C., Drouet C., Lebugle A., Sfihi H., Barroug A. (2007). Nanocrystalline apatites in biological systems: characterisation, structure and properties. Materialwiss. Werkstofftech..

[cit19] Rey C., Combes C., Drouet C., Glimcher M. J. (2009). Bone mineral: update on chemical composition and structure. Osteoporosis Int..

[cit20] Veis A., Dorvee J. R. (2013). Biomineralization mechanisms: a new paradigm for crystal nucleation in organic matrices. Calcif. Tissue Int..

[cit21] Boskey A. L., Villarreal-Ramirez E. (2016). Intrinsically disordered proteins and biomineralization. Matrix Biol..

[cit22] Addison W. N., Azari F., Sorensen E. S., Kaartinen M. T., McKee M. D. (2007). Pyrophosphate inhibits mineralization of osteoblast cultures by binding to mineral, up-regulating osteopontin, and inhibiting alkaline phosphatase activity. J. Biol. Chem..

[cit23] Speer M. Y. (2002). *et al.*, Inactivation of the osteopontin gene enhances vascular calcification of matrix Gla protein-deficient mice: evidence for osteopontin as an inducible inhibitor of vascular calcification in vivo. J. Exp. Med..

[cit24] Kaartinen M. T., Murshed M., Karsenty G., McKee M. D. (2007). Osteopontin upregulation and polymerization by transglutaminase 2 in calcified arteries of Matrix Gla protein-deficient mice. J. Histochem. Cytochem..

[cit25] Brylka L., Jahnen-Dechent W. (2013). The role of fetuin-A in physiological and pathological mineralization. Calcif. Tissue Int..

[cit26] Heiss A., Pipich V., Jahnen-Dechent W., Schwahn D. (2010). Fetuin-A is a mineral carrier protein: small angle neutron scattering provides new insight on Fetuin-A controlled calcification inhibition. Biophys. J..

[cit27] Oldberg A., Franzen A., Heinegard D. (1986). Cloning and sequence analysis of rat bone sialoprotein (osteopontin) cDNA reveals an Arg-Gly-Asp cell-binding sequence. Proc. Natl. Acad. Sci. U. S. A..

[cit28] Steitz S. A. (2002). *et al.*, Osteopontin inhibits mineral deposition and promotes regression of ectopic calcification. Am. J. Pathol..

[cit29] McKee M. D., Nanci A. (1996). Osteopontin at mineralized tissue interfaces in bone, teeth, and osseointegrated implants: Ultrastructural distribution and implications for mineralized tissue formation, turnover, and repair. Microsc. Res. Tech..

[cit30] Bergwitz C., Juppner H. (2010). Regulation of phosphate homeostasis by PTH, vitamin D, and FGF23. Annu. Rev. Med..

[cit31] Yuan Q. (2014). *et al.*, Increased osteopontin contributes to inhibition of bone mineralization in FGF23-deficient mice. J. Bone Miner. Res..

[cit32] Strom T. M. (1997). *et al.*, Pex gene deletions in Gy and Hyp mice provide mouse models for X-linked hypophosphatemia. Hum. Mol. Genet..

[cit33] Brownstein C. A. (2010). *et al.*, Increased bone volume and correction of HYP mouse hypophosphatemia in the Klotho/HYP mouse. Endocrinology.

[cit34] Sitara D. (2006). *et al.*, Genetic ablation of vitamin D activation pathway reverses biochemical and skeletal anomalies in Fgf-23-null animals. Am. J. Pathol..

[cit35] Sitara D. (2008). *et al.*, Genetic evidence of serum phosphate-independent functions of FGF-23 on bone. PLoS Genet..

[cit36] Razzaque M. S., Sitara D., Taguchi T., St-Arnaud R., Lanske B. (2006). Premature aging-like phenotype in fibroblast growth factor 23 null mice is a vitamin D-mediated process. FASEB J..

[cit37] Shimada T. (2004). *et al.*, FGF-23 transgenic mice demonstrate hypophosphatemic rickets with reduced expression of sodium phosphate cotransporter type IIa. Biochem. Biophys. Res. Commun..

[cit38] Bai X., Miao D., Li J., Goltzman D., Karaplis A. C. (2004). Transgenic mice overexpressing human fibroblast growth factor 23 (R176Q) delineate a putative role for parathyroid hormone in renal phosphate wasting disorders. Endocrinology.

[cit39] Glorieux F. H. (2000). *et al.*, Normative data for iliac bone histomorphometry in growing children. Bone.

[cit40] Parfitt A. M., Han Z. H., Palnitkar S., Rao D. S., Shih M. S., Nelson D. (1997). Effects of ethnicity and age or menopause on osteoblast function, bone mineralization, and osteoid accumulation in iliac bone. J. Bone Miner. Res..

[cit41] Edouard T., Glorieux F. H., Rauch F. (2011). Relationship between vitamin D status and bone mineralization, mass, and metabolism in children with osteogenesis imperfecta: histomorphometric study. J. Bone Miner. Res..

[cit42] Cianferotti L. (2022). Osteomalacia Is Not a Single Disease. Int. J. Mol. Sci..

[cit43] Addison W. N., Masica D. L., Gray J. J., McKee M. D. (2010). Phosphorylation-dependent inhibition of mineralization by osteopontin ASARM peptides is regulated by PHEX cleavage. J. Bone Miner. Res..

[cit44] Nakanishi T. (2024). *et al.*, Complex intrinsic abnormalities in osteoblast lineage cells of X-linked hypophosphatemia: Analysis of human iPS cell models generated by CRISPR/Cas9-mediated gene ablation. Bone.

[cit45] Wolf S. E. (2016). *et al.*, Nonclassical crystallization in vivo et in vitro (I): Process-structure-property relationships of nanogranular biominerals. J. Struct. Biol..

[cit46] Rodriguez-Navarro C., Ruiz-Agudo E., Harris J., Wolf S. E. (2016). Nonclassical crystallization in vivo et in vitro (II): Nanogranular features in biomimetic minerals disclose a general colloid-mediated crystal growth mechanism. J. Struct. Biol..

[cit47] Fantner G. E. (2006). *et al.*, Sacrificial Bonds and Hidden Length: Unraveling Molecular Mesostructures in Tough Materials. Biophys. J..

[cit48] Iline-Vul T. (2020). *et al.*, Osteopontin regulates biomimetic calcium phosphate crystallization from disordered mineral layers covering apatite crystallites. Sci. Rep..

[cit49] De Yoreo J. J. (2015). *et al.*, Crystallization by particle attachment in synthetic, biogenic, and geologic environments. Science.

[cit50] Mahamid J., Sharir A., Addadi L., Weiner S. (2008). Amorphous calcium phosphate is a major component of the forming fin bones of zebrafish: Indications for an amorphous precursor phase. Proc. Natl. Acad. Sci. U. S. A..

[cit51] Reznikov N., Bilton M., Lari L., Stevens M. M., Kröger R. (2018). Fractal-like hierarchical organization of bone begins at the nanoscale. Science.

[cit52] Chow W. Y. (2014). *et al.*, NMR Spectroscopy of Native and in Vitro Tissues Implicates PolyADP Ribose in Biomineralization. Science.

[cit53] Baht G. S., Hunter G. K., Goldberg H. A. (2008). Bone sialoprotein-collagen interaction promotes hydroxyapatite nucleation. Matrix Biol..

[cit54] Muller K. H. (2019). *et al.*, Poly(ADP-Ribose) Links the DNA Damage Response and Biomineralization. Cell Rep..

[cit55] Studer D., Humbel B. M., Chiquet M. (2008). Electron microscopy of high pressure frozen samples: bridging the gap between cellular ultrastructure and atomic resolution. Histochem. Cell Biol..

[cit56] Termine J. D., Eanes E. D. (1972). Comparative chemistry of amorphous and apatitic calcium phosphate preparations. Calcif. Tissue Res..

[cit57] Markovic M., Fowler B. O., Tung M. S. (2004). Preparation and Comprehensive Characterization of a Calcium Hydroxyapatite Reference Material. J. Res. Natl. Inst. Stand. Technol..

[cit58] Harmey D. (2006). *et al.*, Elevated skeletal osteopontin levels contribute to the hypophosphatasia phenotype in Akp2(-/-) mice. J. Bone Miner. Res..

[cit59] Wada T., McKee M. D., Steitz S. A., Giachelli C. M. (1999). Calcification of Vascular Smooth Muscle Cell Cultures: Inhibition by Osteopontin. Circ. Res..

[cit60] Addison W. N. (2015). *et al.*, Extracellular matrix mineralization in murine MC3T3-E1 osteoblast cultures: an ultrastructural, compositional and comparative analysis with mouse bone. Bone.

[cit61] Hoac B. (2020). *et al.*, Genetic Ablation of Osteopontin in Osteomalacic Hyp Mice Partially Rescues the Deficient Mineralization Without Correcting Hypophosphatemia. J. Bone Miner. Res..

[cit62] Reznikov N., Almany-Magal R., Shahar R., Weiner S. (2013). Three-dimensional imaging of collagen fibril organization in rat circumferential lamellar bone using a dual beam electron microscope reveals ordered and disordered sub-lamellar structures. Bone.

