# Supplementary material for: Bone mineralization and the effects of elevated osteopontin: from symmetry-breaking foci to 3D space-filling tessellation
Source: Faraday Discuss. 2025 Mar 12;261:406–29. doi: 10.1039/d5fd00013k (PMC12123585; doi:10.1039/d5fd00013k)
Supplement: FD-261-D5FD00013K-s001 [file FD-261-D5FD00013K-s001.pdf]

Supplementary Information (SI) for Faraday Discussions.
This journal is © The Royal Society of Chemistry 2025

Bone mineralization and the effects of elevated osteopontin: From symmetry-breaking foci to 3D space-filling tessellation

Joseph Deering[1], Daniel J. Buss[2], Roland Kröger[3], Hojatollah Vali[2], Maureen J. Lagos[4], Natalie Reznikov[1,2,5] and Marc D. McKee[1,2]

[1] Faculty of Dental Medicine and Oral Health Sciences, McGill University, Montreal, QC, Canada, H3A 0C7

[2] Department of Anatomy and Cell Biology, School of Biomedical Sciences, Faculty of Medicine and Health Sciences, McGill University, Montreal, QC, Canada, H3A 0C7

[3] Department of Physics, University of York, York, United Kingdom, YO10 5DD

[4] Department of Materials Science and Engineering, McMaster University, Hamilton, ON, Canada, L8S 4L8

[5] Department of Bioengineering, Faculty of Engineering, McGill University, Montreal, QC, Canada, H3A 0E9

Corresponding author: Prof. Marc D. McKee
McGill University
Strathcona Anatomy and Dentistry Bldg, Rm M73
3640 University Street
Montreal, QC, H3A 0C7
Canada

Email: marc.mckee@mcgill.ca

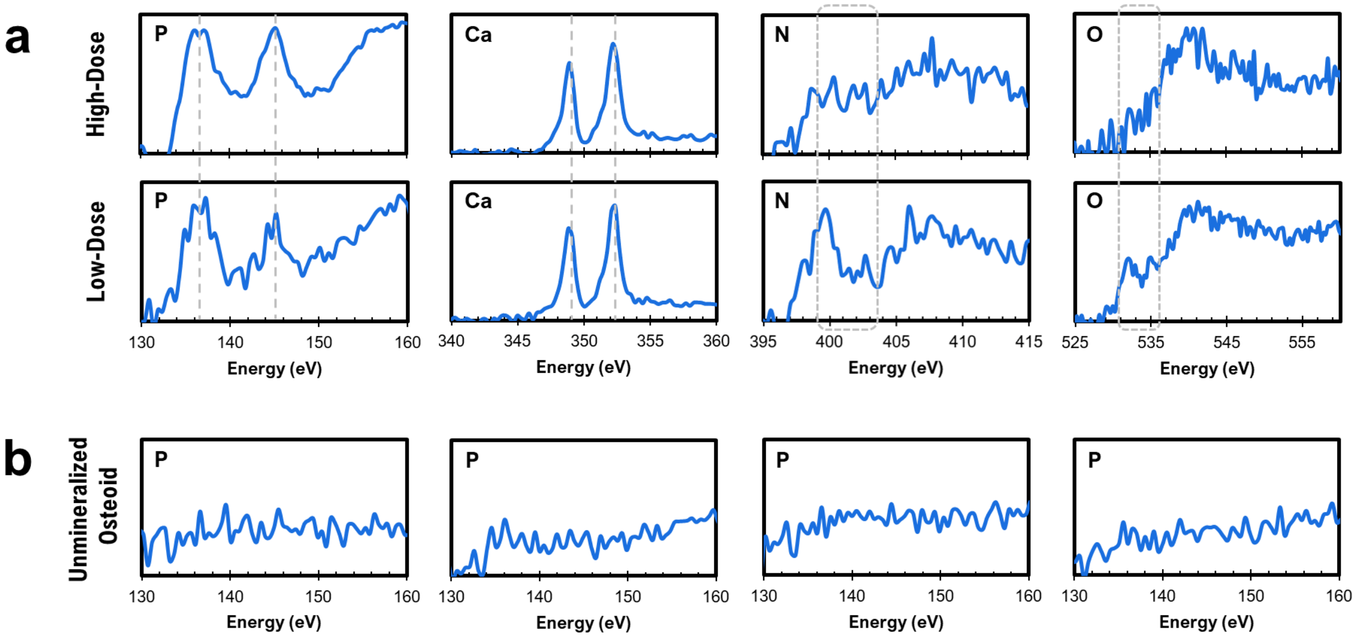

Figure S1: (A) EELS spectra of mineral using comparatively high- and low-dose acquisitions. Peaks are less defined in N and O spectra acquired at high dose (around 402 eV and 534 eV, respectively) while peak shape is well preserved in P and Ca spectra using low-dose acquisition. (B) Phosphorus spectra acquired from unmineralized regions of the ECM. Contributions from organic phosphate in the ECM appear negligible.

Table S1: Repeat Ca/P measurements for three pixels of interest (high, low, and medium Ca/P values) in low-dose EELS spectral image map using different background subtractions. Some changes can be seen on repeat measurement of the same spectra, indicating that the EELS mapping technique can be highly sensitive to noise and the corresponding background subtraction.

	Pixel A	Pixel B	Pixel C
Background Subtraction 1	2.9	1.51	2.29
Background Subtraction 2	2.93	1.92	1.62
Background Subtraction 3	3.2	2.17	1.54
Background Subtraction 4	2.28	1.49	2.86
Background Subtraction 5	2.41	1.3	2.67
Background Subtraction 6	3.28	1.36	2.5
Average	2.83	1.63	2.25
Standard Deviation	0.41	0.34	0.55
